# Alarming Signs and Symptoms in Febrile Children in Primary Care: An Observational Cohort Study in The Netherlands

**DOI:** 10.1371/journal.pone.0088114

**Published:** 2014-02-19

**Authors:** Gijs Elshout, Yvette van Ierland, Arthur M. Bohnen, Marcel de Wilde, Henriëtte A. Moll, Rianne Oostenbrink, Marjolein Y. Berger

**Affiliations:** 1 Department of General Practice, Erasmus MC, Rotterdam, the Netherlands; 2 Department of General Pediatrics, Erasmus MC – Sophia Children's Hospital, Rotterdam, the Netherlands; 3 Department of Medical Informatics, Erasmus MC, Rotterdam, the Netherlands; 4 Department of General Practice, University Groningen, University Medical Center Groningen, Groningen, the Netherlands; University of South Florida, United States of America

## Abstract

**Context:**

Febrile children in primary care have a low risk for serious infection. Although several alarming signs and symptoms are proposed to have predictive value for serious infections, most are based on research in secondary care. The frequency of alarming signs/symptoms has not been established in primary care; however, in this setting differences in occurrence may influence their predictive value for serious infections.

**Objective:**

To determine the frequency of alarming signs/symptoms in febrile children in primary care.

**Design:**

Observational cohort study. Clinical information was registered in a semi-structured way and manually recoded.

**Setting:**

General practitioners' out-of-hours service.

**Subjects:**

Face-to-face patient contacts concerning children (aged ≤16 years) with fever were eligible for inclusion.

**Main outcome measures:**

Frequency of 18 alarming signs and symptoms as reported in the literature.

**Results:**

A total of 10,476 patient contacts were included. The frequency of alarming signs/symptoms ranged from n = 1 (ABC instability; <0.1%) to n = 2,207 (vomiting & diarrhea; 21.1%). Of all children, 59.7% had one or more alarming signs and/or symptoms. Several alarming signs/symptoms were poorly registered with the frequency of missing information ranging from 1,347 contacts (temperature >40°C as reported by the parents; 12.9%) to 8,647 contacts (parental concern; 82.5%).

**Conclusion:**

Although the prevalence of specific alarming signs/symptoms is low in primary care, ≥50% of children have one or more alarming signs/symptoms. There is a need to determine the predictive value of alarming signs/symptoms not only for serious infections in primary care, but as well for increased risk of a complicated course of the illness.

## Introduction

Even though most febrile illnesses in children are harmless, serious infections (e.g. pneumonia, urinary tract infection, meningitis and sepsis) do occur. Predicting the presence of a serious infection is a challenge for the primary care physician. Exploring the presence of so-called alarming signs or symptoms is their most important tool for triage. Alarming signs/symptoms are signs often observed in children with a serious infection and therewith associated with serious infections. Of the many studies investigating the value of alarming signs/symptoms in identifying a serious infection [1], [2] the vast majority were performed in secondary care [3]. A recent study demonstrated that the associations between these alarming signs and serious infections were either weaker or were absent in primary care-datasets, as compared to associations found in secondary care populations [4]. Possible reasons for this lack of association includes differences in patient populations, the way a serious infection is diagnosed [5], that alarming symptoms may also occur in children with viral self-limiting infections, or that primary care studies may include serious infections with a mild prognosis.

Nevertheless, until now the general practitioner's (GP) management is usually guided by the presence of alarming signs/symptoms. The alarming signs/symptoms published in the Dutch guideline and the NICE guideline for the assessment of a febrile child in primary care are based on research performed in secondary care only [6], [7]. However, it is important to determine how frequently these alarming signs/symptoms occur in febrile children presenting in primary care. Our hypothesis was that alarming signs/symptoms frequently occur in this setting. If this is correct, this finding contradicts a strong relation between alarming signs and serious infections because of the expected low prevalence of serious infections in primary care. Therefore, this study determines the frequency of alarming signs/symptoms in a large population of febrile children attending General Practitioner Cooperative (GPC) out-of-hours services in the Netherlands.

## Methods

### Out-of-hours health care system

Out-of-hours primary care in the Netherlands is organized in large-scale cooperatives, which is comparable with the UK, Scandinavia and Australia [8]–[12]. The GPs rotate shifts at the GPCs, and are therefore generally not familiar with the patients they see. In only 5–10% of all primary care consultations, referral to the emergency department is needed [8], [13]. This is similar to the referral rates in the UK, USA and Canada [14], [15]. In the Netherlands, patients who contact the GPC are triaged by telephone by trained assistants to determine if a face-to-face contact is needed. When the GPC is contacted for a febrile child, the assistant determines if alarming signs are present. If not, the assistant will give a telephone advice. When one or more alarming signs are present, a face-to-face contact is indicated.

### Study design

The study design of this study is previously described in detail [16]. In short, this cohort study used data of face-to-face patient contacts (physical consultations and home visits) of febrile children aged ≤16 years that took place at GPC in Rotterdam-Rijnmond between March 2008 and February 2009 (n = 28,234). We excluded telephone consultations, and repeated contacts within 7 days of the initial presentation concerning the same febrile illness. By doing so, we made a selection of children with a new episode of feverish illness in which a GP has to make management decisions. The selection reflects daily practice. The GPs registered information from telephone triage, patient history, physical examination, diagnostic testing, (working) diagnosis, and treatment or referral is documented (by GPs and physician assistants) as written text lines in a semi-structured data sheet.

### Extraction of relevant clinical signs

Signs and symptoms indicative of a serious febrile illness were derived from one systematic review [1], and two published guidelines on the management of febrile children [6], [7]. We included signs which: 1) had a high predictive value (positive likelihood ratio >5.0 or negative likelihood ratio <0.2), 2) were mentioned in at least two of the three data sources, 3) did not represent a diagnosis, and 4) were not prone to high inter-observer variability (e.g. auscultatory sounds) [17]. Selected, closely-related signs were grouped into a total of 18 alarming signs of serious febrile illness (Appendix 1).

### Frequencies of alarming signs and symptoms over different age categories

In addition to the overall frequencies, we looked for the distribution of alarming signs and symptoms over different age categories, since it has been shown that serious infections occur more frequent in younger children [18]. The age categories were predefined, roughly based on ages when children may indicate more and more about their perceived signs and symptoms.

### Correlated alarming signs and symptoms

We grouped 18 alarming signs and symptoms, which is a substantial amount to look for in febrile children. It is of interest if some alarming signs/symptoms frequently occur together. Therefore, we determined the correlation between the different signs and symptoms.

### Missing data

Since the alarming signs/symptoms were obtained from routinely-collected, semi-structured data, missing values were present for each variable (i.e. not mentioned in the medical record). During a consensus meeting with 1 GP (MB), 2 pediatricians (HM, RO) and two residents [general practice (GE) and pediatrics (YvI)] it was decided to deal with missing values in two different ways: 1) the sign/symptom was believed to be so relevant that, if present, the physician would document it. Consequently, all missing values were interpreted as being absent. This was considered for the variables: ill appearance, ABC instability, unconsciousness, drowsy, inconsolable, cyanosis, shortness of breath, meningeal irritation, (febrile) convulsions, vomiting & diarrhea, dehydration, petechial rash, extremity problems; 2) for the remaining signs/symptoms (parental concern, abnormal circulation, signs of urinary tract infection, temperature ≤40°C, and duration of fever) it was decided that the above statements were not applicable, and the percentages of missing values were therefore reported. Contacts without any information from the GP were excluded.

### Details of ethical approval

This study was reviewed by the institution's medical ethics committee (Medisch Ethische Toetsings Commissie Erasmus MC) and the requirement for informed consent was waived (MEC-2012-378).

### Statistical analyses

Patient characteristics and signs/symptoms were analysed using descriptive statistics. In addition, the frequency of the signs/symptoms were provided for different age categories, and tested for statistical significance using a Chi-square test, or a Fishers' exact test when the cell count was <5 events. Statistical significance was set at p<0.05. Correlations were calculated using Pearson's correlation coefficient in order to determine which signs and symptoms were correlated. A correlation – positive or negative – of r = 0.10 to 0.29 was considered low, r = 0.30 to 0.49 was medium, and r = 0.50 to 1.0 was considered high [19]. Data were analyzed using PASW version 17.0.2. for Windows (SPSS, Inc, Chicago, Ill, USA).

## Results

### Description of the population

**Figure 1:**
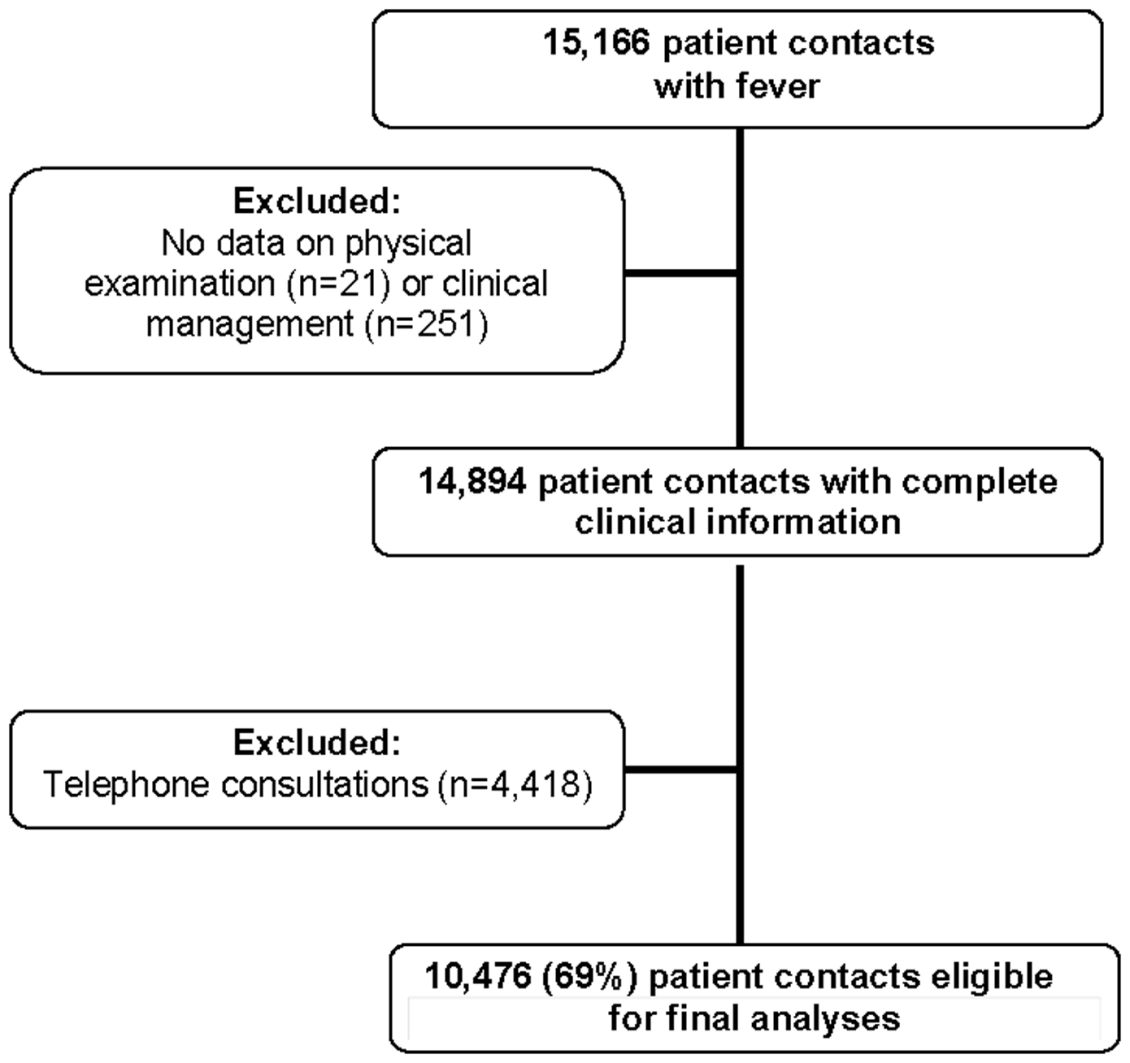
Selection of eligible contacts for the study.

A total of 15,166 patient contacts concerned children with fever. After excluding the telephonic contacts (n = 4,418), and patient contacts with missing data (n = 272), 10,476 patient face-to-face contacts were included in the analysis (Fig. 1). Of these, 5,649 patient contacts concerned boys (53.9%); overall median age was 2.2 (IQR 1.0–4.5) years. Median rectal temperature measured at the GPC was 38.5°C (IQR 37.7–39.1°C). Median duration of fever at time of presentation was 2 (IQR 0–3) days.

**Figure 2:**
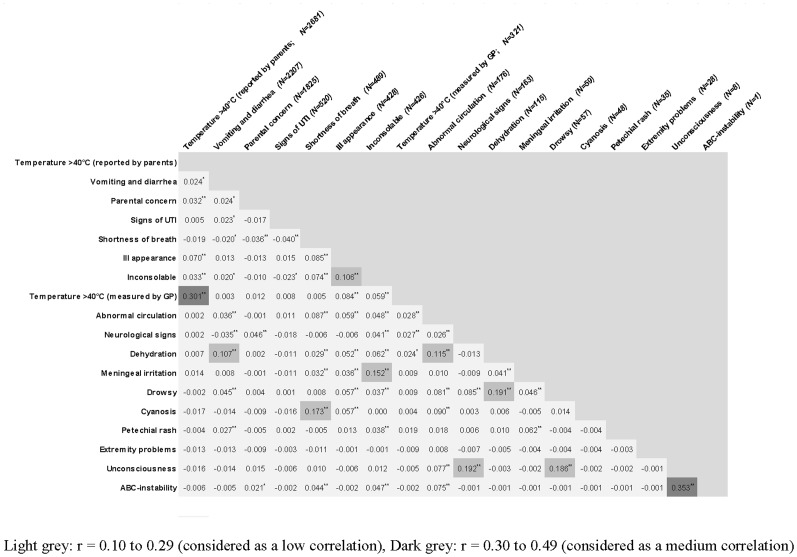
Correlation between alarming signs and symptoms (Pearson's correlation coefficient).

Table 1 presents the frequency of the alarming signs/symptoms per characteristic. The majority of the alarming signs/symptoms were present in ≤10%; vomiting & diarrhea (21%), parental concern (29.2%), temperature >40°C reported by parents (19.8%), and duration of fever >3 days (19.5%) were present in more contacts. Table 2 shows the distribution of the alarming signs/symptoms by age. The presence of one or more alarming signs/symptoms ranged from 49.1–61.6% in the various age categories. Most alarming signs and symptoms are more frequently observed in younger children. Symptoms of UTI were hardly reported under the age of 1 year (0.6%), inconsolable and dehydration are mainly reported under the age of 5 years, abnormal circulation is mostly reported above 12 years. Overall, in 59.7% of the contacts one or more alarming sign/symptom was present (Table 3). Figure 2 shows Pearson's correlation coefficients.; the highest correlations found were a medium correlation between a temperature >40.0C reported by the parents and as measured by the GP (r = 0.301), and a medium correlation between ABC instability and unconsciousness (r = 0.353).

**Table 1:** Presence of alarming signs and symptoms.

Alarming signs and symptoms (N = 10.476)	Present		Absent		Not registered	
	N	Percentage	N	Percentage	N	Percentage of total (N = 10476)
Ill appearance*	428	4.1	10048	95.9	NA	NA
ABC-instability*	1	<0.1	10475	100.0	NA	NA
Unconsciousness*	8	0.1	10468	99.9	NA	NA
Drowsy*	57	0.5	10419	99.5	NA	NA
Inconsolable*	426	4.1	10050	95.9	NA	NA
Cyanosis*	48	0.5	10428	99.5	NA	NA
Shortness of breath*	489	4.7	9987	95.3	NA	NA
Meningeal irritation*	59	0.6	10417	99.4	NA	NA
Neurological signs*	163	1.6	10313	98.4	NA	NA
Vomiting and diarrhea*	2207	21.1	8269	78.9	NA	NA
Dehydration*	115	1.1	10361	98.9	NA	NA
Extremity problems*	28	0.3	10448	99.7	NA	NA
Petechial rash*	35	0.3	10441	99.7	NA	NA
Temperature >40°C (reported by GP)*	321	8.9†	3268	91.1†	6887	65.7
Temperature >40°C (reported by parents)	2681	19.8†	6448	80.2†	1347	12.9
Parental concern	1825	29.2†	4	70.8†	8647	82.5
Abnormal circulation	176	2.4†	2614	97.6†	7686	73.4
Signs of UTI	520	6.0†	3744	94.0†	6212	59.3
Duration of fever >3 days	1589	19.5†	6580	80.5†	2307	22.0

*Assumption: when not mentioned in the record, it is not present.

† Percentage of N registered.

**Table 2:** Alarming signs and symptoms by age category.

	<1 year n = 2609	Percentage	≥1 and <5 years n = 5655	Percentage	≥5 and <12 years n = 1833	Percentage	≥12and ≤16 years n = 379	Percentage
Temperature >40 (stated by parents)* n = 2681	590	22.6	1677	29.7	352	19.2	62	16.4
Vomiting and diarrhoea* n = 2207	642	24.6	1138	20.1	368	20.1	59	15.6
Parental concern* n = 1825	494	18.9	986	17.4	294	16.0	51	13.5
Signs of urinary tract infection* n = 520	15	0.6	281	5.0	193	10.5	31	8.2
Shortness of breath* n = 489	181	6.9	253	4.5	46	2.5	9	2.4
Ill appearance* n = 428	77	3.0	263	4.7	68	3.7	20	5.3
Inconsolable* n = 426	216	8.3	203	3.6	5	0.3	2	0.5
Temperature >40 (measured by GP)* n = 321	70	2.7	203	3.6	42	2.3	6	1.6
Abnormal circulation* n = 176	42	1.6	75	1.3	41	2.2	18	4.7
Neurological signs* n = 163	15	0.6	133	2.4	12	0.7	3	0.8
Dehydration* n = 115	43	1.6	66	1.2	6	0.3	0	0.0
Meningeal irritation* n = 59	33	1.3	16	0.3	7	0.4	3	0.8
Drowsy n = 57	16	0.6	33	0.6	5	0.3	3	0.8
Cyanosis* n = 48	2	0.1	26	0.5	14	0.8	6	1.6
Petechial rash* n = 35	4	0.2	17	0.3	14	0.8	0	0.0
Extremity problems* n = 28	6	0.2	10	0.2	8	0.4	4	1.1
Unconsciousness n = 8	1	0.0	5	0.1	1	0.1	1	0.3
ABC instability n = 1	1	0.0	0	0.0	0	0.0	0	0.0
≥1 alarming sign or symptom* n = 6252	1600	61.3	3484	61.6	982	53.6	186	49.1

*: p-value<0.05.

**Table 3:** Frequency of combined presence of alarming signs and symptoms.

Number of positive alarming signs and symptoms	N	Percentage
0	4224	40.3
1	3837	36.6
2	1711	16.3
3	545	5.2
4	116	1.1
5	31	0.3
6	10	0.1
7	2	0
Total	10476	100

## Discussion

### Statement of principal findings

The frequency of single alarming signs and symptoms for serious infections in febrile children seen by a GP at an out-of-hours service is low; the majority was present in ≤10% of the contacts. However, ≥50% of all children had one or more alarming sign or symptom. These findings are consistent across all age categories. Several signs/symptoms which are expected to be related to serious infections are often poorly registered.

Most alarming signs/symptoms have different frequencies across the age groups. The majority of the alarming signs and symptoms are more frequently seen in younger age groups. However, it has been shown that younger children with fever more frequently suffer from serious infections [18]. Therefore, this finding is not surprising, and does not change our perspective on alarming signs and symptoms for serious infections.

Some alarming signs and symptoms frequently occur together (e.g. unconsciousness and ABC instability); however, none has a relevant correlation with each other. This implies that it is important to look for the presence of every alarming sign and symptom separately, since all are suggested to be related to serious infections and it cannot be assumed that if one sign is absent, the others are also.

### Strengths and weaknesses of the study

A limitation of the study may be that the GPs did not record the clinical signs/symptoms for research purposes. We made the assumption that we can consider some signs (e.g. petechial rash) as being absent when the GP did not specifically report this; however, if this assumption is not correct, the frequency of alarming signs/symptoms will be higher, leading to an even larger discrepancy as compared to the low risk for serious infections in primary care.

Also, because we excluded telephone consultations, our results will overestimate the prevalence of alarming signs/symptoms in febrile children. Nevertheless, assuming that all children with a telephone consultation would have had no alarming signs/symptoms present, the prevalence of children with alarming symptoms is still 41.7%.

In the Netherlands, the out-of-hours service is the only primary care-facility that patients can contact outside regular working hours. This may have led to an overestimation of the alarming sign ‘parental concern’ in comparison to a population presenting during regular hours, since not-concerned parents will be more reluctant to contact the GPC. However, we feel that the described population is representative for a clinically relevant population of febrile children in primary care, since GPs are frequently consulted for febrile children during out-of-hours primary care.

### Findings in relation to other studies

The prediction rules reported in the literature base their predicted risk for a serious infection on multiple alarming signs and symptoms [1]. In the present study, ≥50% of the children had one or more alarming signs/symptoms. This may have a negative effect on the specificity of the prediction rules in predicting serious infections. Since the incidence of serious infections is reported to be low in primary care [18], the frequent occurrence of alarming signs/symptoms will lead to a high false-positive prediction of a serious infection.

### Meaning of the study

In conclusion, the frequency of specific alarming signs/symptoms in primary care is low. However, the proportion of children with more than one alarming signs or symptom is high. Given the high prevalence of alarming signs and symptoms, the low prevalence of serious infections, and the tendency in primary care to actively follow the course of disease in case of diagnostic uncertainty (‘wait-and-see’ management), we suggest that future research on alarming signs and symptoms in primary care should be related to the prognosis of the underlying disease (i.e. hospital admission or duration of complaints), rather than the presence of a serious infection.

## Supporting Information

Table S1 **Grouping of alarming signs into composed determinants of serious infection.**
(DOCX)
