# Supplementary material for: Alarming Signs and Symptoms in Febrile Children in Primary Care: An Observational Cohort Study in The Netherlands
Source: PLoS One. 2014 Feb 19;9(2):e88114. doi: 10.1371/journal.pone.0088114 (PMC3929539; doi:10.1371/journal.pone.0088114)
Supplement: Table S1 — Grouping of alarming signs into composed determinants of serious infection. (DOCX) [file pone.0088114.s001.docx]

**Appendix 1. Grouping of alarming signs into composed determinants of serious infection**

| **Composed alarming signs and symptoms** | **Total selection of alarming signs and symptoms** |
| --- | --- |
| **Parental concern** | Parental concern |
|  |  |
| **Ill appearance** | Clinician’s instinct something is wrong |
|  | Clinically ill appearance |
|  |  |
| **ABCD instability** | ABCD instability |
|  |  |
| **Unconsciousness** | Unconsciousness |
|  |  |
| **Drowsy** | Child is drowsy |
|  | Somnolence |
|  | Reactivity/functional status (decreased) |
|  | Hypotonia |
|  |  |
| **Inconsolable** | Child is inconsolable |
|  | Irritability |
|  | Changed crying pattern |
|  | Child is moaning |
|  |  |
| **Abnormal circulation** | Abnormal skin color (pale, mottled, ashen) |
|  | Capillary refill time > 2 sec |
|  | Tachycardia |
|  |  |
| **Cyanosis** | Cyanosis |
|  | Oxygen saturation <95% |
|  |  |
| **Shortness of breath** | Shortness of breath |
|  | Nasal flaring |
|  | Rapid breathing |
|  | Changed breathing pattern |
|  |  |
| **Meningeal irritation** | Meningeal irritation |
|  | Neck stiffness |
|  | Bulging fontanelle |
|  |  |
| **Neurological signs** | Focal neurological signs |
|  | Paresis/paralysis |
|  | Seizures/fits |
|  |  |
| **Vomiting & diarrhea** | Vomiting (>2x in disease period) |
|  | Diarrhea (>2x in disease period) |
|  |  |
| **Dehydration** | Dry mucous membranes |
|  | Sunken eyes |
|  | Decreased skin elasticity |
|  | Reduced urine output |
|  | Hypotension (APLS) |
|  | Poor feeding |
|  |  |
| **Extremity problems** | Swelling of limb or joint |
|  | Non-weight bearing limb |
|  | Not using an extremity |
|  |  |
| **Signs of urinary tract infection** | Pollakisuria |
|  | Dysuria |
|  | Stomach ache (without other focus for fever) |
|  |  |
| **Petechial rash** | Petechial rash |
|  | Purpura |
|  |  |
| **Temperature ≥40ºC** | Measured at home or at GPC |
|  |  |
| **Duration of fever** | Duration of fever in days at time of consultation |
|  |  |
